# A community perspective on pre-exposure prophylaxis

**DOI:** 10.7448/IAS.17.4.19522

**Published:** 2014-11-02

**Authors:** Simon Collins

**Affiliations:** HIV i-Base, London, UK

## Abstract

The history of pre-exposure prophylaxis (PrEP) is notable for being a community rather than industry-driven development. This talk will review this history, covering factors that include community demand, study results, regulatory challenges, commercial interests and practical issues of public health. It will also look at some of the controversies that appear to limit broader access, and important changes since US approval for PrEP in 2012. If PrEP had been discovered in the 1980s, the demand for access is likely to have been very different and it would now be universally available. Yet in many health settings, the willingness to include the option of PrEP appears to be inversely correlated with the increasingly impressive data showing its effectiveness. The limitations of condoms are shown in continued rates of new HIV infections in high-risk populations. These rates have remained persistently high for the last decade, even with the dramatic impact of treatment as prevention (TasP) on reducing further transmission. Within the last year, the polarized debate about PrEP appears to be shifting to a middle ground focused on individual choice. Together with TasP, this has started a new dialogue on the potential benefits on quality of life. This has brought a new focus on the cumulative and largely unmeasured impact for HIV negative gay men who live for decades focused on a fear of HIV. Looking forwards, the rate-limiting steps of cost and adherence have the potential to be overcome with lower priced generic tenofovir in 2017 and the development of long-acting formulations.

